# Smoker profiles and their influence on smokers’ intention to use a digital decision aid aimed at the uptake of evidence-based smoking cessation tools: An explorative study

**DOI:** 10.1177/2055207620980241

**Published:** 2020-12-29

**Authors:** Thomas Gültzow, Eline Suzanne Smit, Raesita Hudales, Carmen D Dirksen, Ciska Hoving

**Affiliations:** 1 Department of Health Promotion, CAPHRI Care and Public Health Research Institute, Maastricht University, Maastricht, the Netherlands; 2 Department of Communication Science, Amsterdam School of Communication Research/ASCoR, University of Amsterdam, Amsterdam, the Netherlands; 3 Department of Clinical Epidemiology and Medical Technology Assessment, CAPHRI Care and Public Health Research Institute, Maastricht University Medical Center, Maastricht, the Netherlands

**Keywords:** Smoking cessation, decision aid, cluster analysis, decision-making style, smoking, health promotion, health psychology, digital health, decision making

## Abstract

**Objectives:**

Evidence-based smoking cessation support tools (EBSTs) can double the quitting chances, but uptake among smokers is low. A digital decision aid (DA) could help smokers choose an EBST in concordance with their values and preferences, but it is unclear which type of smokers are interested in a digital DA. We hypothesized that smokers’ general decision-making style (GDMS) could be used to identify early adopters. This study therefore aimed to identify smoker profiles based on smokers’ GDMS and investigate these profiles’ association with intention to use a digital DA.

**Design:**

A cross-sectional dataset (N = 200 smokers intending to quit) was used to perform a hierarchical cluster analysis based on smokers’ GDMS scores.

**Methods:**

Clusters were compared on demographic and socio-cognitive variables. Mediation analyses were conducted to see if the relationship between cluster membership and intention was mediated through socio-cognitive variables (e.g., attitude).

**Results:**

Two clusters were identified; *“Avoidant Regretters”* (n = 134) were more avoidant, more regretful and tended to depend more on others in their decision making, while *“Intuitive Non-regretters”* (n = 66) were more spontaneous and intuitive in their decision making. Cluster membership was significantly related to intention to use a DA, with *“Avoidant Regretters”* being more interested. Yet, this association ceased to be significant when corrected for socio-cognitive variables (e.g., attitude). This indicates that cluster membership affected intention via socio-cognitive variables.

**Conclusions:**

The GDMS can be used to identify smokers who are interested in a digital DA early on. As such, the GDMS can be used to tailor recruitment and DA content.

Tobacco smoking continues to be the leading cause of preventable diseases and premature death.^1^ It is estimated that 16% of all deaths in Europe and the Americas can be attributed to smoking.^2^ In the Netherlands, smoking-related mortality is even higher (21%).^2^ Evidence-based smoking cessation support tools (EBSTs) have been shown to be effective in facilitating quitting and maintaining smoking abstinence, i.e., pharmacotherapy, such as nicotine replacement therapy (NRT)^3^; and behavioral support, such as counselling by health professionals.^4^ Such EBSTs more than double successful cessation rates,^5^ but are severely underused^6^ and in the Netherlands uptake of non-evidence based support tools (e.g., acupuncture) is only slightly below that of EBSTs.^7^ EBST uptake could therefore be significantly improved.

However, even if smokers are interested in using an EBST, they still have to choose between all the different EBSTs. It is known that individuals that face health-related decisions often encounter uncertainty about what to choose, which can result in a feeling of discomfort.^8,9^ Offering a decision support system (often called decision aid, DA) to smokers that helps them choose from the multitude of options could help reduce this discomfort.^10^ DAs are specifically designed to facilitate informed decision making between different health-care options, by providing information and helping users to define their own values and preferences regarding these different options.^10,11^ Nowadays, many DAs are delivered online as this allows for a broad and sustainable dissemination.^12–14^ DAs have been used a lot concerning treatment or screening decisions,^10^ and have in a few instances also been used for smoking cessation.^15^ For example, Willemsen et al.^7^ developed a paper-based DA, which was effective in promoting quitting attempts and abstinence. However, it failed to increase uptake of EBSTs and resulted in higher drop-out rates compared to the control group. Early drop-out may cause problems for smokers, as they will not benefit from the intervention,^16^ and for researchers, as this makes it difficult to evaluate the effects.^17^ Designing a digital DA instead of a paper-based DA could potentially already reach more people^12^ and, *particularly* targeting a group that is planning to use a digital DA for EBSTs before it is available (i.e., early adopters),^18,19^ could lead to a more attractive intervention which could prevent high drop-out rates. Such a digital DA aimed at facilitating informed decision making between different EBSTs could be of particular interest for smokers that (1) are planning to stop smoking within the foreseeable future (otherwise, they would probably not be interested in smoking cessation to begin with) and (2) for smokers that hold favorable attitudes towards EBSTs. However, acceptance of or interest in using a DA for lifestyle behavior decisions, such as smoking cessation, by their intended audience has never been assessed (as opposed to interest in using EBSTs, for an example see^5^). DAs especially target processes traditionally linked to deliberate decision making; e.g., the clarification of values through deliberation,^20,21^ which makes them different from traditional health promotion interventions.^10^ However, as people differ in terms of their response patterns to decision-making situations (often called decision-making style),^22,23^ their interest in using an online tool that facilitates deliberative processes could potentially also differ. Therefore, we hypothesize that smokers’ decision-making style may influence their interest in a DA for EBSTs, as not all people value the activation of said cognitive processes targeted by DAs. This information could potentially be used to identify the aforementioned early adaptors. Also, this information could ultimately be used to design a digital DA that is attractive and beneficial to all adopter categories. Researchers usually distinguish five archetypical decision-making styles:^22,23^ (1) *the rational style* is characterized by a thorough search for all information; (2) *the intuitive style* is characterized by a tendency to base decisions on emotions; (3) *the dependent style* is characterized by a tendency to ask for advice and guidance from others; (4) *the avoidant style* is characterized by a general aversion to decision making; and (5) *the spontaneous style* is characterized by people having the tendency go through the decision-making process as quickly as possible.^23^ Other researchers^24^ have added a style which is characterized by post-decisional regret after a decision has been made (i.e., (6) *the regret style*). In general, people do not rely on one style, but rather use a combination of styles.^22^ Therefore, the styles should not be analyzed in isolation. One way to examine the styles and their joint effect on intention to use a smoking cessation DA would be to first identify groups (or clusters) based on decision-making styles and, secondly, to examine whether these groups differ in their intention to use such a DA. As this, to the best of our knowledge, has never been attempted before, we decided to take an explorative approach, leading to our first two research question:

*RQ1:* Is it possible to identify specific groups (or clusters) of smokers planning to quit based on their decision-making styles?

*RQ2:* How do these groups (or clusters) differ in their intention to use a digital DA for EBSTs?

In order to further cross-sectionally validate and compare the identified groups (or clusters),^25^ we decided to compare them demographically and on the basis of smoking (cessation) behavior and other factors that could be associated with the intention of using a smoking cessation DA. Next to the aforementioned favorable attitudes we hypothesize that the following factors could be associated with the intention of using a smoking cessation DA: (1) a smoker’s health locus of control,^26^ as smokers that attribute events in their personal life to either their own or external control as opposed to chance may be more likely to use assistance to achieve their goal^27^ and (2) motivation, as smokers that are planning to stop smoking because it is intrinsically valuable to them (i.e., smokers that are autonomously motivated to stop smoking as defined in the self-determination theory (SDT)^28^) might be more interested in using assistance to achieve said goal (similar patterns have been identified in others domains, e.g.,^29–31^). Autonomous motivation to stop smoking on the other hand has been shown to be related to feelings of perceived competence and perceived autonomy support.^32^ Since no hypotheses could, however, be formulated with regard to differences in clusters in terms of demographics and smoking (cessation) behavior, we formulated the following third research question:

*RQ3:* How do these groups (or clusters) differ in respect to other characteristics, i.e., demographic factors, smoking (cessation) behavior, attitude towards EBSTs, health locus of control, motivation as defined in SDT and factors associated with motivation (i.e., perceived competence and perceived autonomy support)?

## Methods

A cross-sectional study was conducted in January 2018. Participants were recruited through the internet research agency Flycatcher.^33^ Panel members were preselected, based on their smoking status and if they were planning to stop smoking within six months. All preselected participants received an online questionnaire that lasted an average of 15.5 minutes. The questionnaire included an informed consent form, information about EBSTs and information about DAs in general. Participants were excluded if they were younger than 18, did not provide informed consent, or were not planning to quit smoking within six months. In total 250 smokers were invited. Due to the explorative nature of this study, no power analysis has been performed. Therefore, the sample size was based on the experience of the research team. Participants received €1.40 from the internet research agency to complete the questionnaire, which would equal about $1,54.

### Measurements

A Dutch questionnaire was developed and pretested among eight experts and five (ex-)smokers. Pretesting took place with both native and second language Dutch speakers. If possible, validated scales were used that had been used in a Dutch context before. If this was not possible, English validated scales were initially translated by professional translators (forward translation), followed by back-translation by the research team (backward translation).^34^

#### Demographics

Gender identity (0 = woman; 1 = man; 2 = other) and country of birth were measured with one question each, age was measured continuously. Education was measured via three questions. The first question was asked to identify which degree had already been awarded, the second question was asked to identify respondents who were currently enrolled in a degree granting program (0 = no; 1 = yes) and the third to identify which degree granting program they are currently following. Three variables were later transformed: country of origin (0 = Dutch, 1 = Non-Dutch), first and last educational question (0 = low; 1 = medium; 2 = high). No, primary and vocational education were regarded as a low educational attainment; secondary vocational education and a high school degree were regarded as a medium educational attainment and higher vocational education, college and university degrees were regarded as high educational attainment.

#### Smoking behavior and smoking-related cognitions

Smoking behavior assessment was based on the Dutch version of the Fagerström test for nicotine dependence.^35,36^ However, it was adapted to include all smoking behavior, e.g., the original “How soon after you wake up do you smoke your first cigarette?” was changed to “How soon after you wake up do you smoke?” to reflect that not all smokers smoke cigarettes. Vapers (i.e., e-cigarette users) were considered smokers for this study, which was reflected in all measuring instruments, as e-cigarettes are not treated as EBSTs in the Netherlands. The Fagerström test for nicotine dependence had a sufficient level of internal consistency within our sample (α = 0.75). Additionally, used nicotine products (“Which products do you smoke regularly? Multiple answers are possible.”, 0 = cigarette; 1 = hand-rolled cigarette; 2 = e-cigarette; 3 = pipe; 4 = other product) as well as previous cessation attempts (e.g., “How many times have you made a serious quit attempt? By this we mean that you have not smoked for at least 24 hours.”, could be answered on a continuous scale) were examined with four questions based on Mudde et al.^37^ as common in Dutch studies focused on tobacco use/smoking (e.g.,^36^**^,^**^38^**).**

#### Decision-related cognitions

A modified version of the general decision-making style (GDMS) questionnaire^22–24^ was used to assess respondents’ decision-making styles in order to perform a hierarchical cluster analysis. The modified version included questions to measure the regret style mentioned in the introduction. Thus, the scale consists of six subscales each measuring a decision-making style: rational (α = 0.70), intuitive (α = 0.77), dependent (α = 0.81), avoidant (α = 0.86), spontaneous (α = 0.67), and regret (α = 0.79). Answering categories were given on a 5-point scale, ranging from 1 =“strongly disagree” to 5 =“strongly agree”.

#### Other cognitions

The treatment self-regulation questionnaire (TSRQ) was used to assess the degree to which respondents’ motivation for a future cessation attempt is relatively autonomous (autonomous-regulated style) or determined by external control (controlled-regulated style).^39^ The scale consisted of two scales; one measuring the autonomous-regulated style (1 = not at all true, 7 = very true; α = 0.87), one the controlled-regulated style (1 = not at all true, 7 = very true; α = 0.76). Secondly, the perceived competence scale (PCS)^40^ was used to measure how confident respondents felt in their ability to quit smoking^41^ (1 = not at all true, 7 = very true; α = 0.90). Thirdly, the health care climate questionnaire (HCCQ),^40^ was used to capture respondents’ perceptions of the degree to which their health care providers were autonomy-supportive regarding smoking cessation (1 = not at all true, 7 = very true; α = 0.92), i.e., whether they support behaviors that their patients want to do of their own volition. The HCCQ was only administered to respondents that had contact with a health-care professional in the past 12 months.

To assess health locus of control, the multidimensional health locus of control scale (MHLC) was applied to measure the extent to which people believed that they, their physician, or chance have direct control over their own health.^26,42^ The Dutch version of the MHLC consists of three subscales: internal health locus of control (ILOC; α = 0.77), physicians’ health locus of control (PLOC; α = 0.78) and chance health locus of control (CLOC; α = 0.76). Answering categories were given on 6-point scales as originally validated, ranging from 1 =“strongly disagree” to 6 =“strongly agree”.^26^

*Attitude* towards EBSTs was measured with 10 items (5 measuring attitude towards pharmacological support, 5 measuring attitude towards behavioral support) based on Rogers’ Diffusion of Innovation Theory^19^ using a 5-point scale, ranging from 1 =“strongly disagree” to 5 =“strongly agree”. For example, respondents were asked “If I use these tools in a new cessation attempt, I will increase my chances of successfully quitting smoking” for both categories of EBSTs (pharmacological and behavioral support) to reflect Rogers’ relative advantage.^19^ As said items were developed for this study specifically, principal component analyses were conducted before scales were computed. These revealed that one item (based on Roger’s concept of complexity) seemed to measure a different construct. Closer inspection of said item, revealed that it was the only one that was phrased negatively. Recoding of said item, did not change this, which is why we only used the other four items to compute the attitude scales. Both scales showed sufficient alphas (attitude pharmacological support; α = 0.78, and attitude behavioral support; α = 0.79). In-depth results of the principal component analyses can be provided on demand.

Finally, we measured intention to use an EBST DA via three items by asking the respondents whether a hypothetical DA would be used if it was available using a 7-point scale (α = 0.96), e.g., “I want to use an online DA during a next quit attempt (if available).”

### Statistical analysis

All analyses were conducted using SPSS 24.0,^43^ p-values lower than 0.05 were considered significant. Firstly, descriptive analyses were conducted to assess sample characteristics. Secondly, cluster analyses were performed based on the GDMS subscales. Ward’s hierarchical method with Euclidean distance as metric distance was used as clustering algorithm with GDMS scores standardized into z-scores.^44^ Multiple methods were employed to investigate the number of clusters within the sample: (1) cluster profiles were visually inspected to assess shape, level, scatter, and interpretability; (2) inverse scree tests were employed to indicate the optimal number of clusters; and (3) analyses were replicated within three random subsamples to investigate the stability of the cluster solutions.^25,45^

Thirdly, identified clusters were externally validated by comparing them on all measured variables using Mann-Whitney U tests, Kruskal-Wallis H tests or chi-squared tests. For this (and all other analyses that follow) we created a new variable named “cluster membership” based on the cluster analysis described above. Nonparametric tests were used as most variables showed heterogeneity of variance and a substantial number of outliers. For categorical variables chi-square tests were conducted.

Fourthly, we investigated whether cluster membership was associated with intention to use a DA (main outcome variable) by conducting both a simple linear regression (unadjusted) and multiple linear regression (adjusted for other psychological constructs). As no previous studies have investigated the topic at hand before, variables were added to the adjusted model if their respective theoretical backgrounds (e.g., SDT) linked them to decision making and if they were statistically significantly associated with both intention (main outcome variable) and cluster membership (independent variable). A log transformation was applied to the scale measuring the controlled regulated style to not violate the linearity assumption.

Fifthly, mediation analyses were conducted post hoc with the technique as described by Preacher and Hayes^46^ to test for possible mediation to explain the results from the linear regressions. Testing took place for all variables that clusters significantly differed on and/or appeared to be significant in the previous regressions. We tested each possible mediator separately. Results were interpreted using bootstrapped analyses.^46^ As we carried out these mediation analyses in order to better understand the results from the analyses that were planned a priori, they do not correspond to any of the RQs mentioned in the introduction.

## Results

Of the 250 invited smokers, 200 completed the questionnaire and provided informed consent (response rate = 80%). Table 1 displays all sample characteristics.

**Table 1.** Sample characteristics.

**Gender identity**	
Women; n (%)	102 (51.0%)
Men; n (%)	98 (49.0%)
Not listed; n (%)	0 (0.0%)
**Age** Mean (SD)	49.01 (14.25)
**Country of birth**	
The Netherlands; n (%)	191 (95.5%)
Outside The Netherlands; n (%)	9 (4.5%)
**Education level**	
Low (Completed); n (%)	14 (7.0%)
Medium (Completed); n (%)	135 (67.5%)
High (Completed); n (%)	51 (25.5%)
Currently studying for a degree; n (%)	28 (14.0%)
**Smoking behaviour and smoking-related cognitions**	
FTCD; Mean (SD)	3.86 (2.52)
People that had attempted to quit (for at least 24 hours); n (%)	181 (90.5%)
Number of cessation attempts (lasting at least 24 hours); Mean (SD)	3.66 (4.37)
Time in days since last cessation attempt (lasting at least 24 hours); Mean (SD)	957.27 (1528.01)
Duration in days last cessation attempt (lasting at least 24 hours); Mean (SD)	253.33 (676.13)
Cigarette users; n (%)	154 (77.0%)
Hand-rolled cigarette users; n (%)	70 (35.0%)
E-cigarette users; n (%)	24 (12.0%)
Pipe users; n (%)	0 (0.0%)
Other products users; n (%)	7 (3.5%)
**Decision-related cognitions (GDMS)**	
Rational style; Mean (SD)	3.79 (0.61)
Intuitive style; Mean (SD)	4.03 (0.56)
Dependent style; Mean (SD)	3.15 (0.82)
Avoidant style; Mean (SD)	2.86 (0.95)
Spontaneous style; Mean (SD)	3.38 (0.65)
Regret style; Mean (SD)	3.01 (0.89)
**Other cognitions**	
TSRQ: controlled regulatory style; Mean (SD)	3.63 (1.33)
TSRQ: autonomous regulatory style; Mean (SD)	5.55 (1.13)
PCS; Mean (SD)	3.98 (1.37)
HCCQ; Mean (SD)	4.41 (1.18)
Attitude Pharmacological support; Mean (SD)	3.21 (0.98)
Attitude Behavioral support; Mean (SD)	3.09 (0.99)
Internal (health) locus of control; Mean (SD)	18.53 (4.31)
Chance (health) locus of control; Mean (SD)	20.66 (4.45)
Physician (health) locus of control; Mean (SD)	22.98 (4.74)
Intention to use a digital DA for cessation support tools in the future; Mean; (SD)	3.82 (1.76)

Note: Time since last attempt, and duration of the last attempt variables only include people that remembered and tried to quit smoking at least once; FTCD = Fagerström Test for Cigarette Dependence; TSRQ = Treatment Self-Regulation Questionnaire; PCS = Perceived Competence Scale; GDMS = General Decision-Making Style; DA = Decision Aid.

### Cluster analysis

Visual inspection of both the whole and random subsamples’ dendrograms indicated that two clusters were formed, but that those two clusters seemed to split into smaller clusters. Inverse scree tests indicated that the two-cluster solution was best supported by the data, while both the three- and four-cluster-solution seemed feasible. In all three possible solutions, participants differed significantly on most of the GDMS subscales. Comparing the cluster analysis of the whole sample to the cluster analyses of the subsamples showed that clusters replicated most consistently in the two-cluster solution. Based on all available tests, we therefore selected the two-cluster solution for further analysis.

### External validation

The two clusters differed statistically significantly on all of the clustering variables except the rational style. Respondents in cluster 1 (n = 134) indicated that they were more avoidant, dependent and regretful in or after decision-making situations, while respondents in cluster 2 (n = 66) reported a greater tendency to be spontaneous and intuitive. Therefore, cluster 1 was termed *“Avoidant Regretters”*, while cluster 2 was termed *“Intuitive Non-regretters”*. Additionally, *“Avoidant Regretters”* scored significantly higher on the intention to use the digital DA in the future, controlled regulatory style, and both attitude scales. There were also significantly less Dutch people in the *“Avoidant Regretters”* cluster, however 95.5% of the whole sample were born in the Netherlands (therefore, ‘Country of birth’ was not used in further analyses). Examination of the other possible cluster solutions (i.e., three- and four-cluster solutions) revealed that the cluster named *“Intuitive Non-regretters”* remained stable in all of them, while the *“Avoidant Regretters”* seemed to diverge into multiple sub clusters in the other solutions – indicating a more heterogeneous cluster (specific data is available on demand). Table 2 shows the characteristics of the two clusters found in the two-cluster solution.

**Table 2.** Cluster characteristics.

	**Cluster 1 (n = 134)** *Avoidant regretters*	**Cluster 2 (n = 66)** *Intuitive non-regretters*	*p-Value*
**Gender identity**			0.62
Women****; %	52.2%	48.5%	
Men****; %	47.8%	51.5%	
Not listed; %****	0%	0%	
**Age;** Median	49.0	52.0	0.17
**Country of birth**			
The Netherlands; %	93.3%	100%	0.03*
**Education level**			
Low (Completed); %	6.0%	9.1%	0.56
Medium (Completed); %	69.4%	63.6%	0.41
High (Completed); %	24.6%	27.3%	0.69
Currently enrolled in a program; %	17.2%	7.6%	0.06
**Smoking behavior and smoking-related cognitions**			
FTCD; Median	4.0	4.0	0.61
Number of cessation attempts (lasting at least 24 hours); Median	2.0	2.5	0.36
Time (in days) elapsed since last cessation attempt (lasting at least 24 hours); Median	91.31	182.62	0.48
Duration (in days) of last cessation attempt (lasting at least 24 hours); Median	14.0	30.44	0.09
Cigarette users; %	79.9%	71.2%	0.17
Hand-rolled cigarette users; %	34.3%	36.4%	0.78
E-cigarette users; %	12.7%	10.6%	0.67
Pipe users; %	0%	0%	-
Other product users; %	3.0%	4.5%	0.68
**Decision-related cognitions (GDMS)**			
Rational style; Median (Mean rank)	3.8 (97.56)	3.9 (106.47)	0.30
Avoidant style; Median (Mean rank)	3.4 (126.89)	1.9 (46.92)	<0.01*
Intuitive style; Median (Mean rank)	4.0 (84.0)	4.2 (133.99)	<0.01*
Dependent style; Median (Mean rank)	3.4 (123.5)	2.5 (53.81)	<0.01*
Spontaneous style; Median (Mean rank)	3.4 (90.43)	3.6 (120.95)	<0.01*
Regret style; Median (Mean rank)	3.5 (128.84)	2.125 (42.95)	<0.01*
**Other cognitions**			
TSRQ: controlled regulatory style; Median	3.75	3.25	0.03*
TSRQ: autonomous regulatory style; Median	5.5	6.0	<0.01*
PCS; Median	3.75	4.0	0.21
HCCQ; Median	4.08	4.58	0.27
Attitude Pharmacological support; Median	3.25	3.0	0.04*
Attitude Behavioral support; Median	3.25	3.0	<0.01*
Internal (health) locus of control; Median	18.0	19.0	0.65
Chance (health) locus of control; Median	20.0	21.0	0.07
Physician (health) locus of control; Median	22.0	25.0	<0.01*
Intention to use a DA for cessation support tools in the future; Median (mean rank)	4.0 (107.02)	3.5 (87.27)	0.02*

Note: *p < 0.05; FTCD = Fagerström Test for Cigarette Dependence; TSRQ = Treatment Self-Regulation Questionnaire; PCS = Perceived Competence Scale; HCCQ = Health Care Climate Questionnaire; GDMS = General Decision-Making Style; DA = Decision Aid.

### Direct effects of cluster membership

Cluster membership (i.e., if participant belonged to either the *“Avoidant Regretters” or “Intuitive Non-regretters”cluster)* statistically significantly predicted intention to use a digital DA to choose EBSTs, F(1, 198) = 5.06, p = 0.026, accounting for 2.5% of the variation with adjusted R2 = 2.0%. “*Avoidant Regretters”* reported an intention score that was 0.59 points higher than the score of “*Intuitive Non-regretters”*, 95% CI [0.073, 1.108]. In the adjusted multiple regression, cluster membership ceased to statistically significantly predict intention to use a digital DA to choose EBSTs in the future. Table 3 shows the regression model.

**Table 3.** Multiple linear regression identifying determinants of intention to use an EBST DA.

					**Confidence interval**	
**Variable**	**B**	**SE**	**β**	**Sig.**	**Lower**	**Upper**
TSRQ: controlled regulatory style	0.686	0.566	0.075	0.227	0.430	1.802
TSRQ: autonomous regulatory style	0.001	0.098	0.0	0.994	–0.193	0.194
Internal (health) locus of control	–0.063	0.023	–0.155	0.007*	–0.109	–0.018
Physician (health) locus of control	–0.013	0.021	–0.034	0.543	–0.054	0.029
Behavioral Attitude	0.676	0.126	0.381	<0.0005*	0.428	0.925
Pharmacological Attitude	0.482	0.127	0.268	<0.0005*	0.232	0.732
Cluster membership	–0.011	0.219	–0.003	0.959	–0.444	0.421

Note: *p < 0.05; B = unstandardized regression coefficient; SE = Standard error of the coefficient; β = standardized coefficient; DA = Decision Aid; TSRQ = Treatment Self-Regulation Questionnaire.

### Indirect effects of cluster membership

Indications were found that attitude and the controlled regulatory style seemed to fully mediate the effect of cluster membership, while the autonomous regulatory style seemed to mediate the effect only partially. No such indications were found for the internal and physician locus of control scales. Table 4 shows the results of the mediation analyses.

**Table 4.** Mediation analyses.

**Independent variable**	**Mediator**	**Dependent** **variable**	**Mediation** **confidence interval**		**Mediation** **p-value**	**Direct effect ** **of IV on DV** **p-value** **(controlling for M)**
			***Lower***	***Upper***		
Cluster Membership	Behavioral Attitude	Intention	0.2	0.87	<0.01*	0.80
Cluster Membership	Pharmacological Attitude	Intention	0.03	0.67	0.02*	0.28
Cluster Membership	Internal (health) locus of control	Intention	–0.15	0.23	0.66	0.03*
Cluster Membership	Physician (health) locus of control	Intention	0.0	0.35	0.08	0.12
Cluster Membership	TSRQ: controlled regulatory style	Intention	0.14	0.33	0.06	0.09
Cluster Membership	TSRQ: autonomous regulatory style	Intention	–0.36	–0.02	0.045*	<0.01*

Note: *p<0.05; IV = Cluster Membership; DV = Dependent variable; M = Behavioral Attitude, Pharmacological Attitude, Internal locus of control, Physician locus of control, Controlled regulatory style, Autonomous regulatory style.

## Discussion

In this study, it was investigated if clusters of smokers could be identified based on their decision-making styles and how those clusters differed to identify people that were interested in a digital DA for EBSTs.

Two distinct clusters were identified. “*Avoidant Regretters”* were characterized by their avoidant and dependent tendencies, as well as their tendency to regret decisions once those have been made. “*Intuitive Non-regretters”* on the other hand showed a tendency to be spontaneous and intuitive. Although this was the first cluster analysis based on GDMS, correlations between the styles found in earlier studies are comparable to the clusters we found in this study – especially regarding studies that also included regret styles.^23,24^ For example, Dewberry et al.^24^ found moderate to strong correlations between the regret (called brooding in their study), avoidant, and dependent styles and a moderate correlation between the spontaneous and intuitive styles. Furthermore, the authors found no significant correlation between the dependent and the intuitive style.^24^

Additionally, it was found that “*Avoidant Regretters”* showed a significantly higher intention to use a digital DA aimed at facilitating the process of choosing an EBST – corresponding with their prevailing characteristics. Smokers belonging to this cluster showed a higher tendency to avoid decision making in general and also tended to regret decisions after they have been made. DAs are interventions specifically designed to assist decision making^10^ and might consequently be especially appealing to the smokers who might have difficulties with decision making. In addition to their tendency to avoid and regret decisions, “*Avoidant Regretters”* also showed to be more dependent on others when they face decisions. “*Avoidant Regretters”* might perceive a digital DA as an external entity that supports them in their decision making. In line with this reasoning, research by Dewberry et al.^24^ has shown that the dependent, avoidant and regret style are all correlated with the tendency to look for the best option and not to choose the first option that is acceptable (i.e., maximization). While this was not measured in this current study, this might indicate that “*Avoidant Regretters”* prefer strategies aiming at finding the optimal solution, such as using a digital DA.

On the other hand, *“Intuitive Non-regretters”* were characterized by their spontaneity and impulsivity in decision-making situations. Again, two traits that seem to be linked.^23,24^ Our results indicate that individuals in this cluster tend to use emotions to guide decisions and that they prefer quick decisions. Both traits could potentially make traditional DAs less interesting for them, given that they take time to use^10^ and tend to be focused on deliberative thinking.^47^ In line with this reasoning, both styles have not been shown to be linked to maximizing strategies in the study from Dewberry et al.^24^ Therefore, it might be possible that this cluster is less interested to invest time and energy in an intervention that helps them to find the most optimal solutions. And while intuition is sometimes linked to more favorable outcomes such as satisfaction,^48^ previous research has shown the effectiveness of intuition is largely dependent on the level on expertise a person has.^49^ While knowledge or similar constructs were not measured, the two clusters did not differ in terms of their education or their number of cessation attempts. In other words, there is no evidence in this study that *“Intuitive Non-Regretters”* have more smoking cessation or health-related expertise than “*Avoidant Regretters*”. Therefore, even though this type of quitters might be less interested in using a digital DA in the future, they are still likely to profit from it. And in fact, findings indicate that this cluster is interested in the proposed DA, even if they are less interested than “*Avoidant Regretters*” (with an average of 3.5 on a 7-point scale).

Based on the aforementioned findings it could be argued that both clusters could be targeted with a digital DA developed to facilitate choosing an EBST, but that it might be smart to tailor either both the DA itself and/or the recruitment strategy to smokers’ cluster membership. For example, advertisements for the EBST DA could highlight that DAs have been proven to reduce decisional regret. This would probably be especially appealing for “*Avoidant Regretters”*. On the other hand, measures could be taken to make the DA more appealing to *“Intuitive Non-Regretters”*, e.g., by limiting the time needed to use the DA. Integrating intuitive elements^47,50^ in recruitment materials and DA content could also increase the appeal of the DA for *“Intuitive Non-Regretters”*, whereas incorporating proven strategies to reduce regret could increase the attractiveness for “*Avoidant Regretters”*.

While the results of the multivariable regression indicate that the differences in intention may be explained by other variables than cluster membership (such as attitude), mediation analyses show that there is a possibility that the effect of cluster membership on intention is in fact mediated through said variables. While this has not been tested before, this is in line with integrated models that are used to predict behavior (change) in contemporary behavioral science.^51,52^ Those models commonly state that personality characteristics are one of the most distal variables that influence behavior, (partly) mediated through other socio-cognitions. This possibly suggests that belonging to a particular group of decision makers has an impact on socio-cognitions, which in turn influences one’s intention to use a DA.

### Implications for future research

Since our study was explorative in nature and could rather be described as hypothesis-generating (as opposed to hypothesis-testing), efforts should be made to test whether the identified results can be replicated. Ideally, to this end researchers should use a longitudinal design, as our post-hoc results indicate a mediation path may exist from decision-making styles through socio-cognitive variables (e.g., attitude) to intention to use a digital DA. Given the cross-sectional nature of our study, we were unfortunately unable to confirm this mediation path based on the data collected. In view of the so-called “intention-behavior gap”,^53^ it would also be particularly interesting to test whether the identified paths also apply to the actual uptake of a digital DA and not only to the intention to do so. In the discussion we also highlighted a number of ways in which our results can be used to inform either the recruitment of DA participants (e.g., in recruitment materials it could be highlighted that DAs are known to reduce regret) or the (digital) DA design itself (e.g., by including intuitive elements). Researchers could investigate whether suggested recruitment strategies and potential improving alterations to the DA have positive effects (e.g., on DA uptake or on the quality of the decision-making process^54^) and how these effects relate to decision-making styles – e.g., based on our results, one could assume that highlighting regret-reducing in recruitment materials would be especially attractive and effective for *“Avoidant Regretters”*.

### Strength and limitations

This study was mainly limited due to its cross-sectional and explorative nature. As conclusions regarding causality cannot be drawn based on our cross-sectional data, findings should be confirmed by longitudinal data (see also *Implications for future research*). Moreover, due to the explorative nature of our study we were unable to conduct a formal a priori power analysis, limiting confidence in our findings (especially due to our reliance on non-parametric tests which generally require more power). To elaborate, as the sample was relatively small, there is a possibility that we might have missed clusters that would have been identified given a bigger sample. Future research that aims to replicate the findings presented would benefit from conducting an a priori power analysis and recruit a sample of a size required for testing the hypotheses that can be formulated based on these findings. However, despite this limitation we are confident that our findings are valuable, as they reflect modern models and theories and as our findings are in line with findings from comparable research^24^ and as, generally, the sample sizes required to perform the main analysis of this study (i.e., cluster analysis) does not have to be too large.^55^

## Conclusion

Our explorative study has shown that it is indeed possible to identify smoker clusters based on their decision-making styles and that cluster membership has at least some impact on smokers’ intention to use a digital EBSTs DA. Future studies in the field of digital DAs would therefore benefit from including decision-making style in their design, and it would be interesting to see if our findings could be replicated in longitudinal studies. In addition, it would be interesting to see how decision-making style could be used in digital DA development, as well as recruitment of participants for a study on digital DA effectiveness. Practitioners and developers of digital DAs might use our findings to initially tailor their recruitment strategies and identify early adopters, who might be able to contribute to the development of more attractive interventions that are ultimately appealing for smokers from all adopter categories.
